# Alteration of Brain Gray Matter Density After 24 h of Sleep Deprivation in Healthy Adults

**DOI:** 10.3389/fnins.2020.00754

**Published:** 2020-08-11

**Authors:** Jinbo Sun, Rui Zhao, Xuejuan Yang, Hui Deng, Yuanqiang Zhu, Yao Chen, Kai Yuan, Yibin Xi, Hong Yin, Wei Qin

**Affiliations:** ^1^Engineering Research Center of Molecular and Neuro Imaging of the Ministry of Education, School of Life Sciences and Technology, Xidian University, Xi’an, China; ^2^School of Electronics and Information, Xi’an Polytechnic University, Xi’an, China; ^3^Department of Radiology, Xijing Hospital, The Fourth Military Medical University, Xi’an, China

**Keywords:** sleep deprivation, gray matter density, voxel-based morphometry, gray matter volume, cortical thickness, psychomotor vigilance test, sleepiness

## Abstract

It has been reported that one night of acute sleep deprivation (SD) could induce brain structural changes at the synaptic and neuronal levels in animal studies, and could lead to white matter microstructure and cortical thickness change in human neuroimaging studies. In this study, we focused on changes of brain gray matter density (GMD) after one night of acute SD, which has not been explored previously. Twenty-three normal young participants completed the experiment. Each participant underwent twice T1-weighted structural image scanning with one at 08:00 after normal sleep [resting wakeful (RW)] and the other at 08:00 after 24 h of SD. Using voxel-based morphometry (VBM) analysis by FSL-VBM software, we compared GMD between RW and SD. In addition, the gray matter volume (GMV) and cortical thickness (CT) were also calculated based on volumetric and surface measures with FreeSurfer software. The psychomotor vigilance test (PVT) and the Karolinska Sleepiness Scale (KSS) were performed and evaluated for correlation analysis with GMD, GMV, and CT of the significant regions. Our results showed that the GMD in the right frontal pole (FP), right superior frontal gyrus (SFG), and right middle frontal gyrus significantly increased and GMV and CT in the right temporal pole (TP) significantly decreased after 24 h of acute SD. SD-induced changes in GMD in the right middle frontal gyrus were positively correlated with the changes of KSS scores (Spearman’s correlation *r* = 0.625, *p* = 0.0014, Bonferroni correction with *p* < 0.05/25). Taken together, our findings suggested that one night of acute SD could induce substantial brain structure changes and the alterations in GMD in the right middle frontal gyrus (MFG) might be implicated in sleepiness after SD.

## Introduction

Sleep deprivation (SD) has been found to cause functional abnormalities in the human brain (Krause et al., 2017). One night or 24 h of SD can result in significant changes in brain activation which is necessary to perform cognitive tasks, such as working memory, attention, and inhibition control (Chee and Chuah, 2007; Muto et al., 2012; Zhao et al., 2019a). Furthermore, resting-state brain functional activities, such as functional metabolism and functional connectivity, are significantly impacted by acute SD (Akerstedt and Gillberg, 1990; Killgore et al., 2015; Yeo et al., 2015; Xu et al., 2016; Motomura et al., 2017; Zhao et al., 2019b). However, the effect of one night of acute SD on human brain structure has not been thoroughly characterized.

Short-term changes in brain structure after SD have been first observed in animal sleep brain plasticity studies. Previous studies have indicated that sleep is important for cell membranes and myelin maintenance in the brain (Cirelli et al., 2004; Mongrain et al., 2010), and that these structures are susceptible to be damaged by sleep loss (Hinard et al., 2012; Bellesi et al., 2013). Furthermore, recent ultrastructural studies have reported the change of synapse structural plasticity under normal wake and sleep state alternations (Cirelli, 2013; Frank and Cantera, 2014; Areal et al., 2017), the increase of synaptic puncta and spine numbers in fruit flies (Bushey et al., 2011; Liu et al., 2016), and decrease of spine density and dendrite length in rodents after SD (Acosta-Pena et al., 2015; Havekes et al., 2016; Areal et al., 2017). These findings suggest that acute SD may affect brain structure at synaptic and neuronal levels. Besides animal studies, two recent neuroimaging studies have reported significant changes in human white matter and cortical thickness (CT) after one night of acute SD. Using diffusion tensor MRI (DTI), Elvsashagen et al. (2015) found that 23 h of SD caused a remarkable reduction in axial diffusivity, radial diffusivity, and mean diffusivity compared with rested wakefulness (RW) state. Acute SD has also been found to result in the decrease of CT in the bilateral medial parietal cortex (Elvsashagen et al., 2017). Taken together, these animal and human studies indicate that brain structure is susceptible to be changed by acute SD. In addition to white matter integrity and CT, gray matter density (GMD), a structural T1-weighted morphometry measure, is another commonly used index for structural plasticity in neuroimaging measurements (Thomas and Baker, 2013). So far, the impact of SD on GMD is still unclear. Investigation about how GMD is altered after SD would improve our understanding about the brain resistance to sleep loss.

Therefore, in our present study, we explored the effect of 24 h of SD on brain GMD in 23 normal young participants using voxel-based morphometry (VBM). The relationship between GMD change (△GMD) and alterations in vigilant attention [as measured by the psychomotor vigilance test (PVT)] and sleepiness [as measured by the Karolinska Sleepiness Scale (KSS)] after SD were also evaluated. In addition, the gray matter volume (GMV) and CT were also calculated.

## Materials and Methods

### Subjects

The recruitment criteria and experimental procedure were similar to those in our previous researches (Zhu et al., 2017; Zhao et al., 2018). Twenty-five subjects were recruited according to the following inclusion criteria: (1) right handed and healthy; (2) regular sleep schedules of 7–9 h per night, between 22:00 and 08:00; (3) no history of alcohol or drugs abuse; (4) be free of any self-reported medical, psychiatric, neurological, or sleep disorders; (5) not presenting an extreme morning or extreme evening type, assessed by the Munich Chronotype Questionnaire (Chen et al., 2016). One subject was excluded because of the MRI scanner failure. The other subject opted out of this study during the SD session. The final analyzed group consisted of 23 subjects [mean age 20.30 ± 1.64 years; range 17–23; 10 males and 13 females; body mass index (BMI) 21.45 ± 3.25].

All subjects declared that they did not smoke or consume any stimulants, medications, alcohol, or caffeine for at least 24 h before the MRI scanning and provided written informed consent before participation. All research procedures were conducted in accordance with the Declaration of Helsinki and approved by the institutional research ethics committee of the Xijing Hospital of the Air Force Medical University.

### Experimental Procedure

All participants visited the laboratory three times. During the first visit, participants underwent a screening process. They were briefed about the experimental procedure and given instructions about the PVT. Participants were scheduled for the second visit 1 week later. During the second visit, subjects underwent the MR scanning at 08:00 under one of the two states (RW and 24 h of SD) after performing PVT task and rating the sleepiness by KSS. During the third visit, subjects underwent the MR scanning at 08:00 under the other state after KSS and PVT performance. To minimize possible residual effects of SD on cognition (Van Dongen et al., 2003), the state of RW or 24 h of SD was scheduled in a randomized, cross-over fashion with at least 1 week apart. For the SD state, participants were monitored by experimenters and were not allowed to fall asleep from 22:00 to 08:00. They were allowed to engage in non-strenuous activities such as reading and watching videos. For the RW state, subjects were arranged to perform regular sleep. Furthermore, participants were asked to continue their usual daily activities, but were not allowed to engage in shiftwork or stay up all night during the interval between the second and third visits.

### Behavioral Acquisition

We used PVT to measure vigilant attention, which is the cognitive domain most severely impaired by SD (Lim and Dinges, 2008; Yang et al., 2018). This task used in the present study was adapted from our previous research (Zhu et al., 2017, 2018). First, a red fixation cross appeared in the center of a black background on the screen and remained for 2 s. Then, the red fixation cross disappeared, and the black background screen was presented for a random duration of 2–10 s. After that, a red target circle was displayed and participants were instructed to press a button as quickly as possible with their right index finger. They were required to press the button within 30 s. If the participant responded, the red target circle disappeared and the real-time reaction time (RT) was displayed on the screen to provide feedback regarding their performance. The feedback was presented 1 s after the response. If the participant did not respond, the displayed real-time RT was 0 ms. This task lasted for 7 min.

The primary behavioral measurements of interest in the PVT were (1) lapse ratio (the lapse was defined as the trail with RT > 500 ms, and lapse ratio was defined as the number of lapse/trails number); (2) the median RT of all trials; (3) the reciprocal of RT of the fastest 10% trials, labeled “10% fast 1/RT”; and (4) the reciprocal of RT of the slowest 10% trials, labeled “10% slow 1/RT.”

We also assessed sleepiness, the main consequence of insufficient sleep (Akerstedt et al., 2014), using the KSS under RW or SD state. The KSS is a nine-point scale and participants rated sleepiness by circling a number from 1 (very alert) to 9 (very sleepy, fighting sleep) that represented their experience of sleepiness (Akerstedt and Gillberg, 1990).

### MRI Data Acquisition

MRI scanning was performed in a 3T GE MR750 scanner at Department of Radiology, Xijing Hospital, The Air Force Medical University, Xi’an, China. The 3D T1-weighted structural data were obtained using the following parameters: repetition time = 8.2 ms; matrix = 512 × 512; echo time = 3.18 ms; in-plane resolution = 0.5 × 0.5 mm^2^; field of view = 256 × 256 mm^2^; sagittal slices = 196; slice thickness = 1 mm; flip angle = 9°.

### Voxel-Based Morphometry Analysis

The FSL version 5.0.4^1^ was used for VBM analysis (Jenkinson et al., 2012). First, all subjects’ T1-weighted data were brain extracted using the Brain Extraction Tool (BET) (Smith, 2002) and visually checked by an experienced neurologist to remove any leftover non-brain tissue. Second, brain-extracted data were segmented into gray matter, white matter, and cerebrospinal fluid using the FMRIB’s Automated Segmentation Tool (FAST) (Zhang et al., 2001). Third, the obtained gray matter data were non-linearly registered to the gray matter ICBM-152 template using the FMRIB’s Non-linear Image Registration Tool (FNIRT). Fourth, a symmetric, study-specific gray matter template was generated by averaging the registered images. Fifth, the segmented gray matter data of each subject were non-linearly registered to the aforementioned obtained gray matter template and modulated using the Jacobian of the warp field to produce maps of GMD. Finally, the resulting GMD images were smoothed with a Gaussian kernel of 3 mm (a full-width half-maximum of ∼7 mm).

### Cortical Thickness and Volume Analysis

The FreeSurfer longitudinal pipeline version 5.3 was used for brain volume segmentation and cortical reconstruction^2^. Using robust, inverse consistent registration (Reuter et al., 2010), an unbiased within-subject template space and image was created (Reuter and Fischl, 2011; Reuter et al., 2012). Several processing steps such as skull stripping, Talairach transforms, atlas registration, as well as spherical surface maps and parcellations were then initialized with common information from the within-subject template, significantly increasing reliability and statistical power. Then, an additional manual checking step was applied to improve the skull stripping of within-subject templates (see surfer.nmr.mgh.harvard.edu/fswiki/ LongitudinalProcessing for details).

Based on the Desikan/Killiany atlas, the mean value of each volume or thickness segmentation was calculated. For GMV segmentations, 74 left and 74 right cortical gray matter regions, as well as seven left and seven right subcortical gray matter regions were analyzed (for more details, see surfer.nmr.mgh.harvard.edu/fswiki/MorphometryStats). For CT segmentations, 74 left and 74 right cortical gray matter regions were analyzed (for details, see surfer.nmr.mgh.harvard.edu/fswiki/CorticalParcellation).

### Statistical Analysis

In order to explore the effect of SD on the GMD, we compared the GMD maps between RW and SD states using a permutation-based non-parametric paired *t*-test statistical analysis with Threshold-Free Cluster Enhancement (TFCE) correction for multiple comparisons (*p* < 0.05). The significant brain regions were identified according to the Harvard–Oxford cortical and subcortical structural atlas (Desikan et al., 2006). The effect of SD on the GMV and CT was evaluated in 162 and 148 brain regions, respectively. Bonferroni correction was used for multiple comparisons (*p* = 0.05/310). Then, we used the significant brain regions as regions of interest (ROIs) and calculated the GMD, GMV, or CT changes (△GMD, △GMV, or △CT) of these ROIs between these two states (SD-RW). Pearson correlation analysis was performed between △GMD, △GMV, or △CT and the changes (SD-RW) of lapse ratio, median RT, 10% fast 1/RT, and 10% slow 1/RT of PVT. Spearman’s correlation analysis was performed between the △GMD and the △KSS. Age, gender, and BMI were controlled for each correlation analysis. The significant correlation was identified with the Bonferroni correction [*p* < 0.05/(5^∗^*n*); *n* is the number of ROIs and 5 is the number of behavioral measurements].

## Results

### Behavioral Results

**FIGURE 1:**
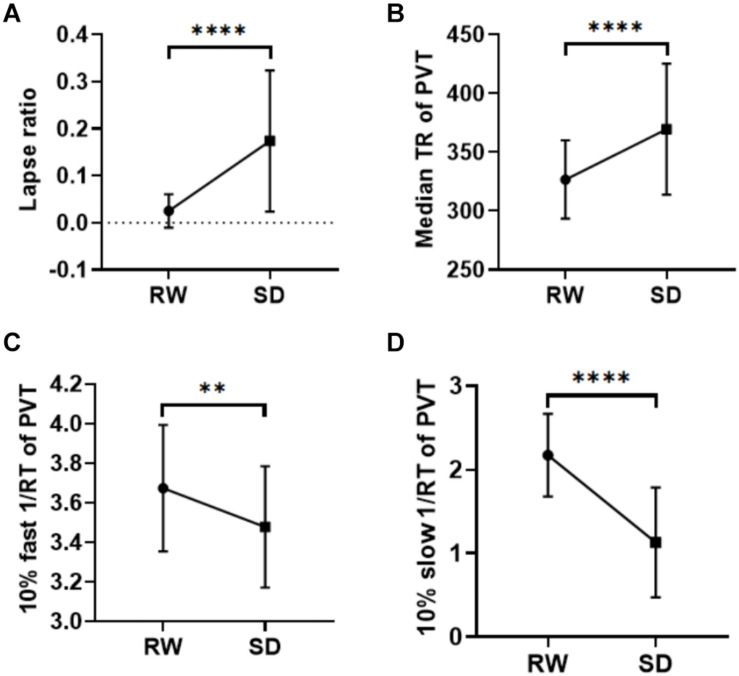
Behavioral results. After SD, participants showed more lapse (*p* < 0.0001, **A**), a slower median RT in the PVT (*p* < 0.0001, **B**), a decreased 10% fast 1/RT (*p* < 0.002, **C**), and reduced 10% slow 1/RT (*p* < 0.0001, **D**). **p < 0.01, ****p < 0.0001. RW, rested wakefulness; SD, sleep deprivation; KSS, Karolinska Sleepiness Scale; PVT, psychomotor vigilance test; RT, reaction time.

After SD, participants exhibited a significant increase in KSS score (Table 1). Poorer performance on the PVT was also observed after SD with increased lapse ratio (*p* < 0.0001), slower median RT (*p* < 0.0001), decreased 10% fast 1/RT (*p* < 0.002), and decreased 10% slow 1/RT (*p* < 0.0001) (Table 1 and Figure 1). The change of each measurement of PVT was not correlated with the change of sleepiness (*p*-values = 0.782, 0.332, 0.387, and 0.654 for lapse ratio, median RT, 10% fast 1/RT, and 10% slow 1/RT of PVT, respectively).

**TABLE 1:** Behavioral changes after sleep deprivation.

	**RW state**	**SD state**	
**Measurements**	**(mean ± SD)**	**(mean ± SD)**	***P*-value**
Lapse ratio of PVT	0.025 ± 0.036	0.174 ± 0.150	<0.0001
Median RT of PVT	326.7 ± 33.3	369.7 ± 55.74	<0.0001
10% fast 1/RT of PVT	3.673 ± 0.319	3.477 ± 0.307	0.002
10% slow 1/RT of PVT	2.172 ± 0.496	1.129 ± 0.657	<0.0001
Sleepiness	3.609 ± 0.783	7.435 ± 1.376	<0.0001

*RW, rested wakefulness; SD, sleep deprivation; SD, standard deviation; PVT, psychomotor vigilance test; RT, reaction time.*

### GMD Changes After SD

**FIGURE 2:**
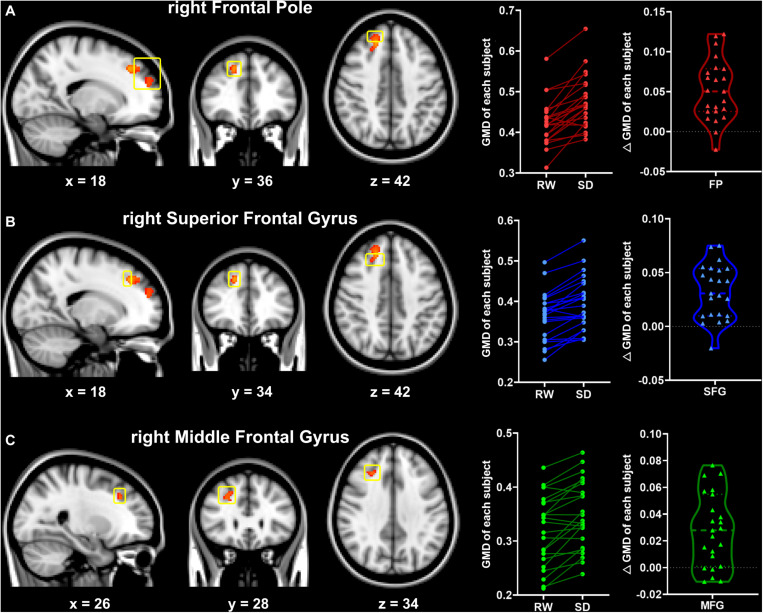
Significant changes in gray matter density between the RW and SD states (RW < SD, TFCE-corrected, *p* < 0.05). Significant brain regions included the right frontal pole (FP, **A**), the right superior frontal gyrus (SFG, **B**), and the right middle frontal gyrus (MFG, **C**). The yellow box indicates the significant brain regions. The numbers at the bottom indicate the MNI coordinates of the slices. RW, rested wakefulness; SD, sleep deprivation.

Significant increase of GMD was observed in several regions (Figure 2) after SD with TFCE correction (*p* < 0.05), including the right frontal pole (FP), the right superior frontal gyrus (SFG), and the right middle frontal gyrus (MFG). There were no regions that exhibited reduced GMD after SD.

### GMV and CT Changes After SD

**FIGURE 3:**
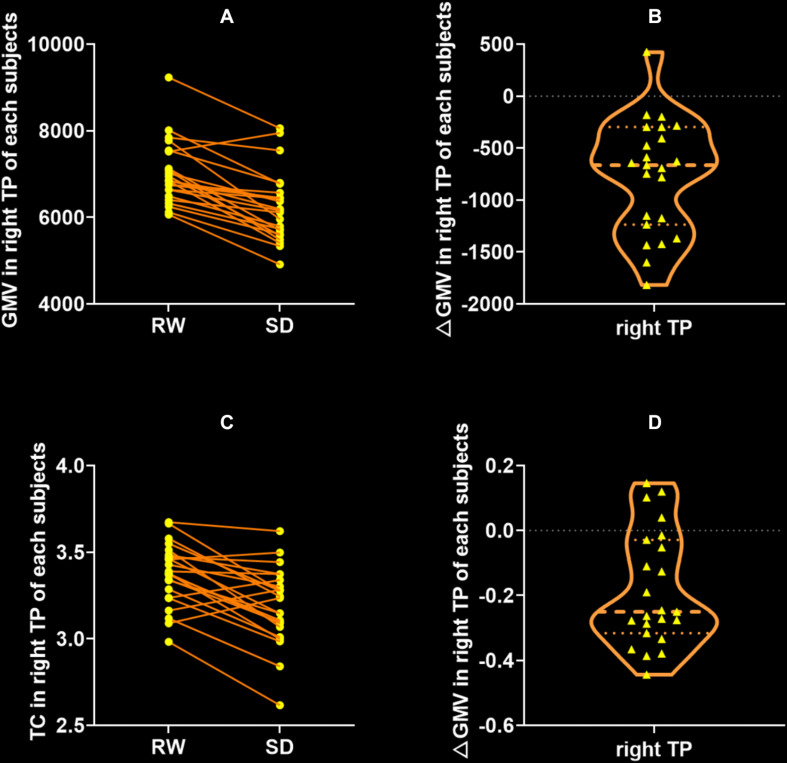
Significant decrease of gray matter volume (GMV) and cortical thickness (CT) between after SD state. The only significant brain region of GMV **(A,B)** and CT **(C,D)** was the right temporal pole (TP). There were no regions that exhibited significant change of GMV or CT after SD. RW, rested wakefulness; SD, sleep deprivation.

Gray matter volume in the right temporal pole (TP) significantly decreased after SD (RW: 7003 ± 728 vs. SD: 6235 ± 806, *t* = 6.714, *p* < 0.0001, Bonferroni correction with *p* < 0.05/310 = 0.00161, Figures 3A,B), and it did not show significant changes in other brain regions. Similarly, CT measurement significantly decreased only in the right TP after SD (RW: 3.37 ± 0.18 vs. SD: 3.19 ± 0.22, *t* = 4.988, *p* < 0.0001, Bonferroni correction with *p* < 0.05/310 = 0.000161, Figures 3C,D).

### Relationship Between Brain Structure Changes and Behavioral Performance

**FIGURE 4:**
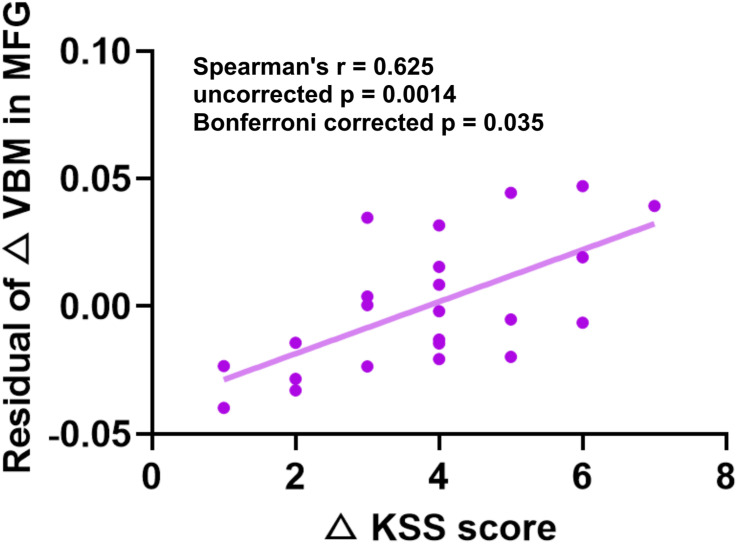
Significant positive Spearman’s correlations between GMD changes in MFG and KSS changes. KSS, Karolinska Sleepiness Scale; GMD, gray matter density; MFG, middle frontal gyrus.

To examine the relationship between the differences between gray structure of the brain and behavioral performance, we correlated △GMD, △GMV, or △CT in the aforementioned significant brain regions with the performance changes of PVT or KSS. Factors of age, gender, and BMI were controlled. All of the correlation results are presented in Table 2. According to the results, △GMD in the MFG and △KSS scores showed significantly positive correlation, as shown in Figure 4 (Spearman’s correlation *r* = 0.625, *p* = 0.0014, Bonferroni correction with *p* < 0.05/25).

**TABLE 2:** Relationship between brain structure changes and behavioral performance.

**Significant ROI**	**Lapse ratio of PVT**		**Median RT of PVT**		**10% fast 1/RT of PVT**		**10% slow 1/RT of PVT**		**Sleepiness**	
	***r*-value**	***p*-value**	***r*-value**	***p*-value**	***r*-value**	***p*-value**	***r*-value**	***p*-value**	***r*-value**	***p*-value**
Right FP (GMD)	–0.1121	0.6107	–0.1171	0.5948	0.4441	0.0338	–0.0201	0.9275	–0.3301	0.2582
Right SFG (GMD)	–0.02273	0.918	–0.132	0.5481	0.3863	0.0686	–0.148	0.5003	0.2939	0.0327
Right MFG (GMD)	0.1836	0.4017	–0.0534	0.8088	0.2724	0.2087	–0.3326	0.121	**0.6351**	**0.0014**
Right TP (GMV)	–0.2224	0.3078	–0.1395	0.5257	0.01877	0.9323	0.056	0.7997	0.07116	0.6808
Right TP (CT)	0.107	0.6271	0.09997	0.6499	–0.3821	0.072	–0.08238	0.7086	0.2183	0.5246

*ROI, region of interest; FP, frontal pole; SFG, superior frontal gyrus; MFG, middle frontal gyrus; TP, temporal pole; GMD, gray matter density; GMV, gray matter volume; CT, cortical thickness; PVT, psychomotor vigilance test; RT, reaction time. Pearson correlations were used between the changes of PVT measurements and brain structure measurements, whereas Spearman correlations were used between the changes of sleepiness and brain structure measurements. Age, gender, and body mass index were controlled for each correlation analysis. The bold values indicated that differences were significant (Bonferroni correction with < 0.05/25 = 0.002).*

## Discussion

In the present study, we investigated the effect of 24 h of SD on GMD using the VBM. We found that GMD in the right FP, the right SFG, and the right MFG significantly increased and that GMV and CT in the right TP significantly decreased after SD. Besides, SD could significantly increase the level of sleepiness and decrease the level of vigilant attention. After controlling factors of gender, age, and BMI, △GMD in the MFG and △KSS scores showed significantly positive correlation. These results suggested that SD could induce alterations in brain structure, which might contribute to the behavioral performance changes.

To our knowledge, this is the first evidence of voxel-wise GMD changes after 24 h of acute SD. Increases of GMD in the right FP, right SFG, and right MFG observed in the study were consistent with Horne’s hypothesis that the PFC was vulnerable to SD (Horne, 1993). The PFC is engaged in attention, working memory, and most of the complex cognitive processes (Whitney et al., 2019). A meta-analytic review of the neural mechanisms underlying vigilant attention identified that the right-lateralized mid- and ventrolateral PFC was one of the core networks engaged during vigilant attention in humans (Langner and Eickhoff, 2013).

Increase of GMD after SD might be related with the Synaptic Homeostasis Hypothesis (Tononi and Cirelli, 2014). As we know, sleep plays a crucial role in the processes associated with learning, memory, and brain plasticity (Diekelmann and Born, 2010; Abel et al., 2013; Rasch and Born, 2013). Recent animal studies have documented the growth of synaptic strength during the waking state, even over a period of a few hours (Bushey et al., 2011). For humans, learning and memory associated with mental and physical activities are common daytime processes and could induce a net increase in synaptic strength in many neuronal circuits while synaptic strength is downscaled to baseline level during sleep (Bushey et al., 2011; Tononi and Cirelli, 2014; Diering, 2017). This downsizing helps sustain energy, make efficient use of gray matter space, and enable synapses to be reused for future memory encoding (Tononi and Cirelli, 2006). However, if participants underwent SD, this downsizing of synaptic strength would be hindered, leading to an increase of synaptic strength oppositely. These changes may further lead to inflation of GMD in the brain macrostructure. Therefore, SD may induce the increase of GMD because of blocking the synapse pruning process.

Sleepiness is the main consequence of sleep loss. Positive correlations between the ΔGMD in the MFG and the changes of sleepiness indicated that participants with greater increase of GMD in the MFG tended to have higher levels of sleepiness after SD. Previous study found that increase in the beta-frequency band power in the MFG was positively associated with sleepiness (Tanaka et al., 2014) and the fatigue score significantly correlated with gray matter atrophy in the MFG in multiple sclerosis (Sepulcre et al., 2009; Fuchs et al., 2019). Thus, the accumulation of daytime activities might be leading to the increase in the beta-frequency band power and synaptic strength in the MFG. Considering the previous evidence that SD might prevent the downscaling of synaptic strength growth to baseline in the PFC and the result of sleepiness increase after SD observed in our study, the correlations between the △GMD in the MFG and the changes of sleepiness in this study might be a reflection of this neural mechanism. What is more, Elvsashagen et al. (2015) have investigated the effect of one night of SD on cerebral white matter DTI parameters and found that widespread axial diffusivity decreased after SD and that larger decreased axial diffusivity was correlated with greater levels of sleepiness after SD. They also found significant cortical thinning in the bilateral medial parietal cortex after SD, and noted that greater thinning was associated with a higher level of sleepiness after SD (Elvsashagen et al., 2017). These results suggest that, besides GMD, sleepiness could also be associated with CT and DTI indices. Further research could explore whether these changes in brain structure represent the neural basis of increased sleepiness after SD.

Besides GMD increase, significant decreases of GMV and CT in the right TP after SD were also observed (Figure 3). As previously reported, the structural and functional changes in TP were associated with the poor sleep quality in normative aging (Amorim et al., 2018) and insomnia (Santarnecchi et al., 2018). The plasticity in the right TP is also involved in the sleep-dependent motor memory (Walker et al., 2005a) and visual skill learning (Walker et al., 2005b). Considering that CT in the bilateral medial parietal cortex significantly decreased after SD (Elvsashagen et al., 2017), the significant decrease in GMV and CT in the TP in the study may reflect the inhibition of sleep-dependent activities induced by acute SD.

The brain structure changes found in this study were induced by acute total sleep loss. Bernardi et al. (2016) have investigated the effect of the intensive, prolonged task training on structural indices. They found decreased cortical mean diffusivity, declined ventricular volume, and increased gray and white matter subcortical volumes after 24 h of task practice combined with SD, but all changes reverted after 8 h of sleep recovery. Therefore, the alterations of GMD, GMV, and CT after acute SD may be neurobiologically temporary effects and could be reverted following 1 day or few days of recovery sleep. However, numerous studies have documented that sleep disorders and psychiatric disorders comorbid with sleep loss are associated with the brain gray matter abnormalities. Previous structural MRI studies have demonstrated the gray matter atrophy/inflation in patients with chronic insomnia (Dang-Vu, 2013; Tahmasian et al., 2018). How acute SD-induced reversible brain structural changes develop into long-term sleep problem-induced irreversible brain structural damage is an important topic to be studied and explored in the future.

Several limitations should be considered when interpreting the present findings. First, it is known that hydration affects brain structure easily (Dickson et al., 2005; Duning et al., 2005; Kempton et al., 2009; Streitburger et al., 2012), which is hard to be avoided. In our experimental design, we asked participants not to deliberately avoid water intake or to drink too much water during SD period and in the morning after normal sleep. Although the impact of hydration on the results could not be completely ruled out, we have minimized the impact of hydration. Besides, because a previous study argued that ventricular volume is more sensitive to dehydration or hyperhydration than GMV (Streitburger et al., 2012), we analyzed the changes in ventricular volume and no significant difference in ventricular volume was observed between the SD and RW periods (*p* = 0.178). Taken together, we believed that the changes in GMD in this study were unlikely caused by hydration. Second, we only compared the GMD after normal sleep with that after 24 h of SD and found significant brain structure changes after SD. However, the recovery effect on these short-term brain structure changes is unclear. Bernardi et al. (2016) reported that brain structure could be restored after recovery sleep. Further studies are required to explore the restoration effect of sleep recovery on brain structure. Third, recent studies have reported that behavioral performance and task-related neural responses were dynamically changed during SD (Muto et al., 2016; Zhu et al., 2018, 2019). Further studies should investigate the dynamic changes in GMD during one night of SD. Fourth, the sample size in the present study was relatively small. Our findings should be verified in a larger sample. Finally, one night of SD can cause substantial deterioration of multiple types of cognitive performance. We only examined the relationship between GMD changes, PVT performance, and sleepiness. Further research should take other types of cognitive performance into consideration.

## Conclusion

We identified increased GMD in the PFC and decreased GMV and CT in the TP after SD, and found a relationship between the △GMD in the MFG and the changes of sleepiness. These findings indicate that the brain gray matter structure is vulnerable to acute SD, and that alterations in GMD may be implicated in sleepiness after SD.

## Data Availability Statement

The datasets generated for this study are available on request to the corresponding author.

## Ethics Statement

The studies involving human participants were reviewed and approved by the Institutional Research Ethics Committee of the Xijing Hospital of the Air Force Medical University. The patients/participants provided their written informed consent to participate in this study.

## Author Contributions

JS, YX, HY, and WQ were guarantors of integrity of the entire study. JS, YZ, and WQ contributed to study concepts/study design. JS, YX, and HY contributed to data acquisition. JS, YC, and RZ contributed to data analysis/interpretation. JS, RZ, XY, and HD contributed to manuscript drafting or manuscript revision. All authors contributed to manuscript revision and read and approved the submitted version.

## Conflict of Interest

The authors declare that the research was conducted in the absence of any commercial or financial relationships that could be construed as a potential conflict of interest.

## Footnotes

1 http://www.fmrib.ox.ac.uk/fsl/

2 http://surfer.nmr.mgh.harvard.edu/
